# Goodbye genome paper, hello genome report: the increasing popularity of ‘genome announcements’ and their impact on science

**DOI:** 10.1093/bfgp/elw026

**Published:** 2016-06-22

**Authors:** David Roy Smith

**Keywords:** genome announcement, genome paper, genome report, genome sequencing, preprint servers, scientific publishing

## Abstract

Next-generation sequencing technologies have revolutionized genomics and altered the scientific publication landscape. Life-science journals abound with genome papers—peer-reviewed descriptions of newly sequenced chromosomes. Although they once filled the pages of *Nature* and *Science*, genome papers are now mostly relegated to journals with low-impact factors. Some have forecast the death of the genome paper and argued that they are using up valuable resources and not advancing science. However, the publication rate of genome papers is on the rise. This increase is largely because some journals have created a new category of manuscript called genome reports, which are short, fast-tracked papers describing a chromosome sequence(s), its GenBank accession number and little else. In 2015, for example, more than 2000 genome reports were published, and 2016 is poised to bring even more. Here, I highlight the growing popularity of genome reports and discuss their merits, drawbacks and impact on science and the academic publication infrastructure. Genome reports can be excellent assets for the research community, but they are also being used as quick and easy routes to a publication, and in some instances they are not peer reviewed. One of the best arguments for genome reports is that they are a citable, user-generated genomic resource providing essential methodological and biological information, which may not be present in the sequence database. But they are expensive and time-consuming avenues for achieving such a goal.

## Birth of the genome paper

Nearly four decades ago, Sanger *et al.* [1] decoded, for the first time, the entire DNA sequence of a genome, that of the bacteriophage ΦX174. This historic achievement also marked the inception of a new genre of scientific article: the genome paper. It would take another 4 years before scientists sequenced and published a human chromosome—our mitochondrial genome [2]—and an additional 15 years for the arrival of a nuclear genome paper (yeast) [3]. These and other pioneering genome sequences, such as that of *Haemophilus influenzae* [4], had a massive and lasting impact on life-science research. The human mitochondrial genome paper [2], for example, has been cited more than 8000 times.

By the turn of the millennium, genome papers were fast becoming among the most publicized and cited articles within the scientific literature. The simultaneously published articles describing the human genome [5, 6] topped Thomson Reuters’ *Science Watch* list of ‘hot papers’ in biology of 2001 [7]. These same articles were also widely covered by scientific and popular news media, which has become a recurring theme for genome papers of all stripes. Just think of all the university press releases, journal editorials and news stories that you have read highlighting the publication of a genome sequence, many with formulaic titles like ‘Genome of … gives insight into the evolution of …’

Genome papers also mirror the shift in genetic research from small group efforts—think Watson and Crick or Beadle and Tatum—toward large international collaborations and giant consortiums. The mouse genome paper [8] boasts of >200 authors from >40 different institutes. Many lead authors of landmark genome papers have gone on to become world-renowned researchers, in some cases winning the Nobel Prize (e.g. Frederick Sanger) or achieving celebrity status (e.g. Craig Venter), reinforcing the influence that genome sequencing and genome papers have had on science, society and culture. Indeed, the race (and the ensuing soap opera) to sequence and publish the draft human genome has been detailed in various bestselling books, including Venter’s autobiography *A Life Decoded, My Genome: My Life* [9]. And, more recently, books like *Postgenomics: Perspectives on Biology after the Genome* [10] have begun to explore the political and historical impacts of sequencing the human genome.

The technological breakthroughs brought about by the human genome project resonated throughout the research world and helped usher in a new age of automated capillary Sanger sequencing [11]. By the mid-2000s, even small laboratory groups were sequencing and publishing articles on entire chromosomes, particularly those from viruses, bacteria and eukaryotic organelles; and large consortiums were publishing nuclear genome papers every few months or sooner. Publishers responded to the growing popularity of genome sequencing by creating an ever-increasing number of journals specializing in genomic data. Online-only open-access journals in particular, including *Genome Biology*, *BMC Genomics*, *PLOS ONE* and *DNA Research*, have become popular outlets for genome papers, as have many traditional in-print journals (e.g. *Molecular Genetics and Genomics*).

The advent of massively parallel next-generation sequencing (NGS) technologies [12] and sophisticated user-friendly bioinformatics software [13] eventually brought genomics (and the potential to publish a genome paper) to the scientific masses. As one commentary in *Nature Methods* aptly put it: ‘With the publication of more than 100 research articles in less than two years, next-generation sequencing has demonstrated its enormous potential for anyone working in the life sciences … [and] has brought the field of genomics back into the laboratories of single investigators or small academic consortia, as is evidenced by the fact that the majority of next-generation sequencing publications originate from sites other than the large genome centers’ [14]. As massively parallel sequencing methods took hold, the scientific community’s response was an incessant stream of genome papers from just about every chromosome and organism imaginable. If it could be sequenced and assembled, it was packaged and sold as a genome paper.

A glance at the enormous growth of GenBank over the past half-decade [15], particularly the Sequence Read Archive [16], which houses trillions of trillion base pairs of NGS data, underscores the immense influence that high-throughput sequencing has had on genomics and science as a whole. NGS has helped bring the number of completely sequenced genomes (and associated genome papers) to staggering heights. As of 1 April 2016, GenBank contains  >60 000 prokaryotic genomes and  >2700 eukaryotic nuclear genomes. But by far the most highly sequenced eukaryotic chromosomes are mitochondrial and plastid DNAs (mtDNAs and ptDNAs) (>7600). Not surprisingly, organelle genomes are also among the most publicized type of chromosome, giving rise to >2600 genome papers in the past 5 years [17]. This flood of genome sequence information has greatly improved our understanding of genetics and provided an inexhaustible reservoir of data for comparative studies, but it has also deflated the importance of genome papers.

## Genome paper overkill

As online sequence repositories and journals swell with DNA data, the novelty and scientific worth of genome papers have waned [18, 19]. Like most trends in research, cutting-edge approaches can quickly become dull and commonplace. No longer does the sequencing of a nuclear genome guarantee a publication in a prestigious journal. Although, admittedly, the potential for hype still exists, as recently demonstrated by the tardigrade and seagrass nuclear genome papers [20, 21], which appeared in *PNAS* and *Nature*, respectively, and received considerable media coverage. However, both of these papers reinforce the notion that today only the most compelling genomes find their way into top journals. In the case of the tardigrade genome, the authors argued that an astoundingly large number of genes (∼6000) were horizontally acquired [20]—a finding that was subsequently disputed [22].

Exceptions aside, most contemporary genome papers, especially those of viruses, bacteria, mitochondria and plastids, are relegated to small journals with low impact factors. As a researcher studying organelle genomic architecture, I can attest that it is getting much harder to publish mtDNA and ptDNA articles, even in relatively specialized journals. In confronting these challenges, and in the hopes of increasing and broadening the impact of their studies, some researchers have started describing many different genomes sequences in a single paper—the multi-genome paper [23, 24]. Others are now skipping the publication step entirely and just depositing their newly sequenced genomes in GenBank and leaving it at that. The latter strategy, despite helping the progression of science, provides little payoff for the authors; unlike peer-reviewed papers, GenBank entries are not the standard currency for obtaining university degrees, promotions or research grants. In the publish-or-perish academic landscape, the journal article is king. Moreover, GenBank data does not provide details of why the sequence was generated or background information about the organism and its habitat [19].

No matter where (or if) it gets published, a genome paper can be an excellent contribution to science. Organelle genome data, for instance, are used in a range of disciplines, including medicine, archeology, forensics and evolutionary biology, and therefore the articles describing these data can be widely read, highly cited and scientifically relevant [17]. Organelle genomes can also have irregular architectures and unconventional modes of expression that are unparalleled in other genetic compartments [25]. In the euglenozoan *Diplonema papillatum*, for example, the *cox1* gene is transcribed from nine different mitochondrial chromosomes, giving nine partial transcripts that are joined via trans-splicing and then translated using a nonstandard genetic code [26]. The characterization of complicated genomes, like the *D. papillatum* mtDNA, typically requires an assortment of experiments and detailed results, making these types of DNAs well suited for genome papers and poor candidates for the GenBank-only approach to genomics. Moreover, GenBank entries have strict formatting rules, which do not permit comprehensive information about the methods and results, and thus cannot, by themselves, easily convey the architecture of complex genomes—and that is to say nothing about the poor level of gene annotation of some GenBank data and the absence of peer review.

Asset or not, genome papers are undoubtedly tying up valuable scientific resources. As publication rates of genomic data soar, more and more journal editors and referees are spending their time reviewing genome papers, and an increasing number of scientists are investing their energy in writing them. Over the past year, I have been invited to review >25 organelle genome papers, and I have also written and submitted four of my own for publication. Some contemporary genome papers address fundamental scientific questions and exemplify the best of what genomics has to offer. But others, arguably, are unoriginal, formulaic, add little in terms of new knowledge and are potentially distracting scientists from more valuable tasks [17]. The publishers of these papers, however, are not complaining. Authors can pay anywhere from a few hundred to a few thousand dollars to see their sequenced genomes in print. From 2010–2015, more than 2000 mitochondrial genome papers or genome reports (defined below) were published; if one conservatively estimates an average publication fee of US $500 per article, then that means more than 1 million dollars (of mostly taxpayer money) was spent on mtDNA papers. Factor in all the other types of chromosomes and genome papers are a big business—but are they about to go bust?

Given the diminishing impact of newly sequenced genomes and that the scientific literature is saturated with articles describing them, one could be forgiven for thinking that the end of the genome paper is in sight. Indeed, various researchers have predicted the death of the genome paper and have pointed out the many flaws of a ‘sequence-first-ask-questions-later’ approach to genomics [18, 27, 28]. Some of these sentiments were summarized eloquently by Viney [28] in a Science & Society article for *Trends in Parasitology*: ‘We have to recognise the paucity of knowledge and understanding on which our genomics analyses are based. The failure of genomics is all of our failure, for not thinking critically about what we really understand, and what we can only infer. Genomics has not yet delivered for biology’. Although in the same issue of the journal Wasmuth [29] wrote in defense of genomics: ‘… it is not surprising that several opinion pieces have recently been published calling into question the value of genomics research and of the genome sequence data. With respect to parasites, the questions are: has our understanding of parasite biology been improved [by genomics], and how does this affect disease control? The answers are, undoubtedly, yes and greatly.’

Perhaps Hall [27] described the genomic era best in his essay *After the Gold Rush*:

We, that is genomicists, have been spoiled. We have been real-estate agents working in a housing boom; bankers trading in debt. We have not been made to work; worse still, there has been very little incentive to think. … we have constantly been fed a high-calorie diet of technical improvements that have led to startlingly obvious (but interesting) discovery experiments to perform; experiments that were impossible or too costly only months previously. What do you do after you have sequenced a human genome? Sequence 1000 human genomes! When you have done that, sequence 2000 human genomes, sequence their microbiomes, sequence their transcriptomes, sequence Earth. These are all sensible things to do, the only reason they had not been carried out before is because they couldn’t. Many (but not all) genomic experiments are not ingenious or elegant, they are brute force discovery projects made possible by clever technology. The technology has been doing the thinking for us. But, as with all exponential trends in ecosystems or economies, the party always has to come to an end.

But for the genome paper, despite predictions, the party has not come to an end; it has only grown larger, noisier and more crowded, and it is now much faster to gain entry. This ease of admission is not only owing to technological improvements but also because certain journals have a created a new fast-tracked and stripped-down version of the genome paper often referred to as the genome report.

## Rise of the genome report

Faced with an increasing number of articles describing DNA data and a need for more appropriate venues to present these data, some publishers and journals have responded by changing the structure and format of genome papers. Specifically, certain journals have started accepting very short manuscripts (500–1500 words) that present a new chromosome sequence, its GenBank accession number and little else. These pint-sized articles go by various names, such as genome reports, genome announcements, genome notes or genome letters (Table 1), but will be referred to here broadly as genome reports. Their short length and minimal number (or complete absence) of figures, tables and article subheadings are a significant departure from long-form genome papers, which typically span 8–10 journal pages, contain many supporting items and have formal introduction, methods, results and discussion sections.

**Table 1.** Examples of journals that currently publish genome-report-type articles

Journal name	Publisher	Article type	Peer reviewed?^a^	Impact factor^b^	Description (taken directly from journal webpage)
Genome Announcements	American Society for Microbiology	Genome announcement	No	N/A	A 500-word report stating that the genome of a particular organism (prokaryote, eukaryote or virus) has been sequenced and providing a citable record of the corresponding GenBank submission.Must include abstract but no text headings can be used except for ‘Acknowledgments’ and ‘References’.Cannot include figures, tables or supplemental material to present data or analysis.
Genome Biology and Evolution	Oxford University Press	Genome report	Yes	4.2	Focused 1500-word papers (up to six tables or figures) that publish the main evolutionary message of new genome sequences as they become submitted to GenBank.May also contain specifically focused comparative analyses of previously published genomes that contain a substantial and novel insight of broadest evolutionary significance.
Journal of Biotechnology	Elsevier	Genome announcement	No	2.9	A 500-word report announcing the availability of the completely annotated genome sequence of a biotechnologically relevant organism in the corresponding database (for eukaryotes, advanced draft genomes will also be considered).Articles can contain an Abstract, a brief report on the organism and its biotechnological relevance, a table summarizing the genome features, References and an Acknowledgement. Figures are generally not allowed.
Journal of Genomics	Ivyspring	Genome note	Yes	N/A	A 1000-word report (10 reference limit; conclusions not permitted) describing novel data sets from high-throughput analysis of genotypes, phenotypes, gene expression, metabolomes, proteomes or genome assemblies.Standard metrics for data quality and the experimental design must be clearly reported.
Memórias do Instituto Oswaldo Cruz	Oswaldo Cruz Foundation	Genome announcement and highlight	Yes	1.6	Dedicated to publishing new genome information from eukaryote parasites, virus, bacteria and their respective vectors, as well as re-sequencing or comparative genome analyses.Should occupy no more than three printed pages including figures and/or tables.
Mitochondrial DNA^c^	Taylor & Francis	Mitogenome announcement	Yes	1.2	A 500-word report describing a newly sequenced organelle genome with no subheadings except for abstract (100–200 words), main text and references, and one figure, which must be a phylogenetic tree.
Molecular Ecology Resources	Wiley	Genomic resources note	No	3.7	Short notes on newly assembled and annotated transcriptomes, genome fractions or whole genomes, and/or a library of SNP/SSR markers.Authors submit a short manuscript describing how the resource was developed and where the data can be accessed.Do not appear in journal as individual papers but are instead published as part of a summary article.
Standards in Genomic Science	BioMed Central (Springer)	Short genome report	Yes	3.2	Short (∼500-word) article on newly sequenced genome. Article format must follow guidelines and template (available from journal Web site) put forward by the SGS.Any manuscripts not using template or that are missing key figures, tables and/or references (as per the guidelines) will be returned to authors.Rationale of the content model is to provide information that is consistently and uniformly presented for rapid and easy consumption by both human and machine readers.

^a^Editorial review was not considered peer review.

^b^Most recent impact factor; taken from journal Web site.

^c^As of 1 January 2016, the journal *Mitochondrial DNA* has been split into two sister journals: *Mitochondrial DNA Part A: The Journal of DNA Mapping, Sequencing, and Analysis* and *Mitochondrial DNA Part B: Resources*—mitochondrial genome announcements are published in the latter [31].

Reputable journals that currently publish genome reports (alongside other kinds of papers) include *Genome Biology and Evolution* (Oxford University Press), *Molecular Ecology Resources* (Wiley), *Standards in Genomics Science* (Springer) and *The Journal of Biotechnology* (Elsevier), to name but a few (Table 1). The American Society for Microbiology (ASM) recently started a new online-only open-access journal called *Genome Announcements* dedicated to publishing 500-word notes (with no figures or subheadings) on the availability of recently sequenced prokaryotic, eukaryotic and viral genomes. Similarly, the journal *Mitochondrial DNA* (Taylor & Francis) publishes mitogenome announcements, which are 500-word reports (with a single figure) on newly sequenced organelle genomes [31, 32].

Despite their diminutive size, genome reports are generally no cheaper to publish than standard research articles. As of 1 April 2016, publication fees for the journal *Genome Announcements* are US $560 per article (for non-ASM members), which translates to about 1 dollar per word. Publishing a genome report in the journal *Standards in Genomics Science* is more expensive, costing £890 (∼US $1265), which is ∼US $2.5/word. Article-processing charges are higher still for a genome report in *Genome Biology and Evolution*: US $1800, but this comes with a 1500-word limit, which is triple that of most other journals. For all of these examples, the open-access fees are included in the price, as are color-figure charges, when figures are permitted.

Similar to the article-processing fees, the publication rates of genome reports tend to be high. For example, *Standards in Genomics Science* published 89 genome reports throughout 2015. In the same year, *The Journal of Biotechnology* published 97 genome reports, and *Mitochondrial DNA* released more than 500 mitogenome announcements. The publication rates are even greater for *Genome Announcements*, which contained an astonishing 1330 genome reports in 2015, amounting to more than half a million dollars in article processing costs. Altogether, well over 2000 genome reports appeared in the past year alone, and based on the preliminary publication rates for 2016, this year is poised to bring even more. In addition to demonstrating the popularity of genome reports, these high rates of publication are also a reflection that many (but not all) of the journals publishing these types of papers are online-only and thus not constrained by page-number limits or printing costs.

In some cases, genome reports are drowning out other categories of article within the journals in which they appear. Recently, the lead editor of *Mitochondrial DNA* noted: ‘The increase of genome announcements at *Mitochondrial DNA* is interesting … and supports [the] contention that there is an inordinate rise in these kinds of reports. From 2009 to the present the rise in percent of announcements in the journal [went] from 50% to 80%. The percentage of pages in the journal dedicated to announcements [rose] from 25% to 50% over the same period’ [33]. In short, half the journal has been consumed by genome reports. The editor goes on to say: ‘This steep incline in interest in publishing [mitogenome] announcements by researchers has prompted the editors at mtDNA to create a “resources” publication specifically for genome announcements’ [33]. In other words, the number of genome reports submitted to *Mitochondrial DNA* has gotten so large that the editors have created a new open-access journal titled *Mitochondrial DNA Part B: Resources* that is catered to genome reports and other short technical reports [30].

The creation of journals devoted to genome reports is a logical response to the fast-changing world of genomics research, and one that could benefit the scientific community. In 2008, the Genomics Standards Consortium (GSC)—an organization promoting the implementation of genomic standards [34]—came out in favor of genome reports:

The scientific community is in the midst of a publishing revolution … marked by a growing shift away from a traditional dichotomy between ‘journal articles’ and ‘database entries’ and an increasing adoption of hybrid models of collecting and disseminating scientific information. With respect to genomes … we feel the scientific community would be best served by the immediate launch of a central repository of short, highly structured ‘Genome Notes’ that must be standards compliant. This could be done in the context of an existing journal, but we also suggest the more radical solution of launching a new journal. [35]

Less than a year later, the GSC launched the journal *Standards in Genomics Science*, which publishes genome reports that meet the requirements outlined by the GSC. These requirements include ‘minimum information’ features, such as sequencing and annotation methods, which can be housed in tightly integrated databases, like the Genomes OnLine Database, and be easily read by both humans and machines [35]. The ultimate goals of the GSC and their associated journal are for genomic information to be stored in a citable, concise and uniform manner outlining how and why the sequence was generated and including details about the source organism [19, 35].

The GSC also believes that genome reports should be centralized to a single journal, Web site or database, thus maximizing the benefits to the research community [35]. This is a sound objective, but unfortunately it is one that has not yet been achieved. Many journals publish genome reports and in some instances these journals have different formatting and data requirements, which do not necessarily meet those put forward by the GSC. Complicating things further is that genome reports can be published under different names (e.g. genome announcements), meaning that they cannot be easily or quickly accessed, searched or compared from a central databank. For example, using the PubMed advanced search platform, I was unable to easily distinguish mitogenome announcements from other types of articles published in *Mitochondrial DNA*. If the future of genomic data dissemination is dependent on genome reports, then it will be imperative that authors, journals, publishers and the International Nucleotide Sequence Database Collaboration work together to make these reports accessible and comparable across platforms. The GSC has done a great job at developing the ground rules for how this cooperation can take place.

## The pros and cons of genome reports

There are many reasons why the scientific community should embrace genome reports, some of which have been outlined by the GSC [19, 34, 35]. Again, one of the best arguments for genome reports is that they are a citable, peer-reviewed and user-generated genomic resource providing essential methodological and biological information, which may not be present in the sequence database. Moreover, if constructed in a concise and consistent manner and following sound guidelines, genome reports allow genomic data to be quickly and easily compared by people and computers. Genome reports are also short, meaning that unlike full-length papers they take up less space in printed journals and are fast to write up and read, saving time for authors, editors and reviewers. They are also better suited to projects that are focused on generating new DNA sequence data rather than those addressing a specific biological question (e.g. the evolution of multicellularity). Most importantly, genome reports, with some exceptions, give researchers peer-reviewed credit for sequencing, assembling and annotating genomes.

But there are reasons why the scientists might want to move away from genome reports. One big drawback is that they are expensive to publish, ranging from US $1–2/word. If every future genome sequence is to be accompanied by a genome report, then tens of millions of research dollars will be spent on article-processing fees. Although great for the publishing industry, these funds could probably be spent on more meaningful scientific endeavors. The GSC has compared the rise of user-generated genomic information, including genome reports, to Flickr and Wikipedia, which have given the world millions of freely available images and articles, respectively [34]. But unlike publishing a genome report, uploading an image to Flickr or writing an article for Wikipedia is free. If genome reports are to succeed, organizations like the GSC will likely have to develop or champion a cheaper or free publication format.

Another potential shortcoming of genome reports is that they are not necessarily peer reviewed. Manuscripts submitted to *Genome Announcements*, instead of being sent to anonymous referees for feedback, are reviewed by the editor(s) alone and if deemed acceptable are published shortly after submission. *Molecular Ecology Resources* employs a similar review process for genome reports. Other journals, however, including *Genome Biology and Evolution*, use a more conventional peer-review protocol. If one of the key benefits of genome reports is to provide researchers with citable, peer-reviewed genomic resources, then those resources should meet the appropriate standards of scientific peer review, and if they do not then they should not be listed as peer-reviewed content on a curriculum vita or anywhere else.

If peer review is not a prerequisite, then open preprint servers may be a good alternative to journals for storing and disseminating genome reports. Preprint servers are permanent, open databanks that house research articles without the condition that those articles undergo peer review, editorial oversight or typesetting before publication (articles are screened for non-scientific content and plagiarism) [36, 37]. Popular preprint servers that currently accept genomics-related articles include arXiv, bioRxiv, PeerJ PrePrints, figshare and ResearchGate [36]. There are many reasons why preprint servers are ideal for genome reports: (1) Preprints go up shortly after submission allowing for fast dissemination of unpublished work; (2) They are free to access and most have no publication fees; (3) Preprint articles are citable via a digital object identifier and are indexed by some academic search engines (e.g. Google Scholar); (4) readers can comment on articles, providing a level of peer review; (5) there are no limitations on word length or figure/table number; and (6) articles can be updated and revised, even years later. A quick scan of bioRxiv reveals that genome reports have already started appearing on preprint servers [38, 39].

## Conclusion

Whether they are published as research articles, short reports or preprints, or sent directly to GenBank, genome sequence data are here to stay. The shift away from genome papers to genome reports reflects much broader trends in how life-science research is marketed and published. Many journals now publish short reports, including *Science*, *The Journal of Cell Biology* and *Current Biology.* There are also a number of journals that publish biological data sets of any sort. The Nature Publishing Group recently started the journal *Scientific Data*, which specializes in the publication of data sets that may not be suitable for traditional publication outlets. Like the journals publishing genome reports, *Scientific Data* is founded on the principle that scientists who invest in making data sets widely available and reusable deserve appropriate credit and recognition.

Some journals have stepped away from publishing genome reports; as of 1 January 2015, *FEMS Microbiology Letters* no longer publishes genome announcements. It is important to remember as well that there was a time when high-profile journals would publish papers describing a newly sequenced gene. Then there was a phase when gene-sequence papers, like genome papers, existed as short reports, but now they cannot even be found in preprint servers. Like the gene sequence paper, the ultimate destiny of the genome paper, genome report, genome announcement or whatever title and form it takes, might be extinction. Perhaps the genome report is the beginning of the end for the publication of genome sequences—or maybe it is just getting going.

## Funding

This work was supported by a Discovery Grant to DRS from the Natural Sciences and Engineering Research Council (NSERC) of Canada.
